# 6,6′-Dimeth­oxy-2,2′-[pyridine-2,3-diylbis(nitrilo­methyl­idyne)]diphenol

**DOI:** 10.1107/S160053680905020X

**Published:** 2009-12-09

**Authors:** Qiuhong Li

**Affiliations:** a College of Materials Science and Engineering, Shandong University of Technology, Zibo 255049, People’s Republic of China

## Abstract

In the title compound, C_21_H_19_N_3_O_4_, two intramolecular N—H⋯O hydrogen bonds generate two six-membered rings. The dihedral angles between the central heterocyclic ring and the two pendant rings are 61.5 (2) and 63.5 (1)°.

## Related literature

For related crystal structures, see: Cimerman *et al.* (1992 ▶); Bi *et al.* (2007 ▶).
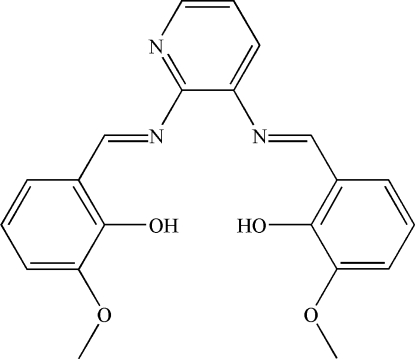


## Experimental

### 

#### Crystal data

C_21_H_19_N_3_O_4_
*M* *_r_* = 377.39
Monoclinic, 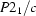

*a* = 6.7006 (9) Å
*b* = 16.699 (2) Å
*c* = 17.490 (2) Å
β = 98.772 (2)°
*V* = 1934.2 (5) Å^3^
*Z* = 4
Mo *K*α radiation
μ = 0.09 mm^−1^
*T* = 293 K
0.25 × 0.21 × 0.19 mm

#### Data collection

Bruker APEXII CCD area-detector diffractometer
Absorption correction: multi-scan (*SADABS*; Sheldrick, 2008*a* ▶) *T*_min_ = 0.978, *T*_max_ = 0.983
9149 measured reflections
3293 independent reflections
1929 reflections with *I* > 2σ(*I*)
*R*_int_ = 0.039

#### Refinement

*R*[*F* ^2^ > 2σ(*F* ^2^)] = 0.060
*wR*(*F* ^2^) = 0.184
*S* = 1.04
3293 reflections
257 parameters
H-atom parameters constrained
Δρ_max_ = 0.40 e Å^−3^
Δρ_min_ = −0.44 e Å^−3^

### 

Data collection: *APEX2* (Bruker, 2004 ▶); cell refinement: *SAINT-Plus* (Bruker, 2001 ▶); data reduction: *SAINT-Plus*; program(s) used to solve structure: *SHELXS97* (Sheldrick, 2008*b* ▶); program(s) used to refine structure: *SHELXL97* (Sheldrick, 2008*b* ▶); molecular graphics: *SHELXTL* (Sheldrick, 2008*b* ▶); software used to prepare material for publication: *SHELXTL*.

## Supplementary Material

Crystal structure: contains datablocks I, global. DOI: 10.1107/S160053680905020X/hg2604sup1.cif

Structure factors: contains datablocks I. DOI: 10.1107/S160053680905020X/hg2604Isup2.hkl

Additional supplementary materials:  crystallographic information; 3D view; checkCIF report

## Figures and Tables

**Table 1:** Hydrogen-bond geometry (Å, °)

*D*—H⋯*A*	*D*—H	H⋯*A*	*D*⋯*A*	*D*—H⋯*A*
O1—H1⋯N1	0.82	1.96	2.683 (3)	146
O3—H3⋯N2	0.82	1.85	2.568 (3)	146

**Fig. 1.**
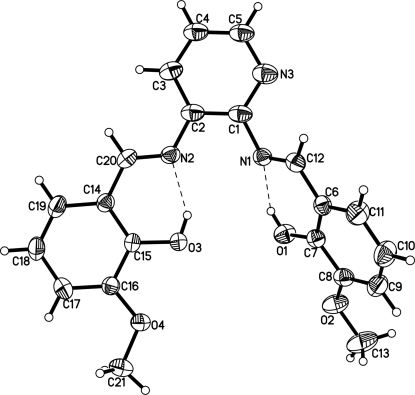
View of the title compound with the atom-labelling scheme. Displacement ellipsoids are drawn at the 30% probability level.

## supplementary crystallographic information

### Comment

Transition metal complexes with salen-type Schiff-bases as ligands, which has been known as one of the oldest ligands in coordination chemistry, have been intensely studied. Here, we report a new Schiff-base ligand based on 2,3-diaminopyridine and 2-hydroxy-3-methoxybenzaldehyde.

The geometry and labeling scheme for the crystal structure of the title compound are depicted in Figure 1. As can be seen from Figure 1, the title compound affords the potentially tetradentate ligand contributed from the O_2_N_2_ donor unit. The imide bond lengths are 1.267 (4)Å for N1—C12 and 1.288 (4)Å for N2—C20, respectively, which are basically consistent with the corresponding distances found in other diaminopyridine-based Schiff base ligand (Cimerman, *et al.*, 1992; Bi *et al.*, 2007). There exists relative strong intramolecular O-H···N hydrogen bonding in this compound with the N···O distance 2.683 (3)Å and the bond angle 145.8 ° for O1—N1 and 2.568 (3))Å , 146.1° for O3—N2, respectively; and is similar to its analogues above mentioned.

### Experimental

The title Schiff base ligand was synthesized by condensation 2,3-diaminopyridine and 2-hydroxy-3-methoxybenzaldehyde with the ratio 1:2 in ethanol. Single crystals suitable for X-ray diffraction were obtained after the solvent was partially evaporated.

### Refinement

All the H atoms were placed using the HFIX commands in *SHELXL-97*, with C—H distances of 0.93, 0.96 Å and O—H 0.82 Å, and were allowed for as riding atoms with *U*_iso_(H) = 1.5*U*_eq_(C), *U*_iso_(H) = 1.2*U*_eq_(C) and *U*_iso_(H) = 1.2*U*_eq_(O), respectively.

### Crystal data

**Table:**

C_21_H_19_N_3_O_4_	*F*(000) = 792
*M**_r_* = 377.39	*D*_x_ = 1.296 Mg m^−^^3^
Monoclinic, *P*2_1_/*c*	Mo *K*α radiation, λ = 0.71073 Å
Hall symbol: -P 2ybc	Cell parameters from 1473 reflections
*a* = 6.7006 (9) Å	θ = 2.4–22.1°
*b* = 16.699 (2) Å	µ = 0.09 mm^−^^1^
*c* = 17.490 (2) Å	*T* = 293 K
β = 98.772 (2)°	Needle, yellow
*V* = 1934.2 (5) Å^3^	0.25 × 0.21 × 0.19 mm
*Z* = 4	

### Data collection

**Table:**

Bruker APEXII CCD area-detector diffractometer	3293 independent reflections
Radiation source: fine-focus sealed tube	1929 reflections with *I* > 2σ(*I*)
graphite	*R*_int_ = 0.039
φ and ω scans	θ_max_ = 24.9°, θ_min_ = 1.7°
Absorption correction: multi-scan (*SADABS*; Sheldrick, 2008a)	*h* = −7→7
*T*_min_ = 0.978, *T*_max_ = 0.983	*k* = −16→19
9149 measured reflections	*l* = −18→20

### Refinement

**Table:**

Refinement on *F*^2^	Primary atom site location: structure-invariant direct methods
Least-squares matrix: full	Secondary atom site location: difference Fourier map
*R*[*F*^2^ > 2σ(*F*^2^)] = 0.060	Hydrogen site location: inferred from neighbouring sites
*wR*(*F*^2^) = 0.184	H-atom parameters constrained
*S* = 1.04	*w* = 1/[σ^2^(*F*_o_^2^) + (0.0889*P*)^2^ + 0.4093*P*] where *P* = (*F*_o_^2^ + 2*F*_c_^2^)/3
3293 reflections	(Δ/σ)_max_ = 0.001
257 parameters	Δρ_max_ = 0.40 e Å^−^^3^
0 restraints	Δρ_min_ = −0.44 e Å^−^^3^

### Special details

**Table:**

Geometry. All e.s.d.'s (except the e.s.d. in the dihedral angle between two l.s. planes) are estimated using the full covariance matrix. The cell e.s.d.'s are taken into account individually in the estimation of e.s.d.'s in distances, angles and torsion angles; correlations between e.s.d.'s in cell parameters are only used when they are defined by crystal symmetry. An approximate (isotropic) treatment of cell e.s.d.'s is used for estimating e.s.d.'s involving l.s. planes.
Refinement. Refinement of *F*^2^ against ALL reflections. The weighted *R*-factor *wR* and goodness of fit *S* are based on *F*^2^, conventional *R*-factors *R* are based on *F*, with *F* set to zero for negative *F*^2^. The threshold expression of *F*^2^ > σ(*F*^2^) is used only for calculating *R*-factors(gt) *etc*. and is not relevant to the choice of reflections for refinement. *R*-factors based on *F*^2^ are statistically about twice as large as those based on *F*, and *R*- factors based on ALL data will be even larger.

### Fractional atomic coordinates and isotropic or equivalent isotropic displacement parameters (Å^2^)

**Table:**

	*x*	*y*	*z*	*U*_iso_*/*U*_eq_	
O1	0.0595 (3)	0.84140 (12)	0.30034 (13)	0.0651 (6)	
H1	0.0164	0.8097	0.3297	0.098*	
O2	0.1593 (4)	0.95259 (14)	0.20681 (15)	0.0796 (8)	
O3	0.2804 (3)	0.84262 (13)	0.49519 (12)	0.0597 (6)	
H3	0.1757	0.8164	0.4888	0.090*	
O4	0.6184 (3)	0.91857 (13)	0.53308 (12)	0.0627 (6)	
N1	−0.1950 (4)	0.78816 (15)	0.39342 (14)	0.0500 (6)	
N2	0.0306 (4)	0.73504 (13)	0.52610 (15)	0.0521 (6)	
N3	−0.4678 (4)	0.69480 (18)	0.41075 (18)	0.0764 (9)	
C1	−0.2765 (5)	0.72718 (19)	0.4364 (2)	0.0616 (9)	
C2	−0.1580 (5)	0.69766 (19)	0.5039 (2)	0.0609 (9)	
C3	−0.2348 (6)	0.63538 (19)	0.5445 (2)	0.0692 (10)	
H3A	−0.1586	0.6151	0.5892	0.083*	
C4	−0.4230 (5)	0.6040 (2)	0.5184 (2)	0.0695 (10)	
H4	−0.4725	0.5626	0.5457	0.083*	
C5	−0.5373 (6)	0.6328 (2)	0.4533 (2)	0.0688 (10)	
H5	−0.6641	0.6108	0.4369	0.083*	
C6	−0.2355 (4)	0.91289 (17)	0.32671 (16)	0.0498 (7)	
C7	−0.0614 (4)	0.90562 (17)	0.29041 (16)	0.0484 (7)	
C8	−0.0096 (5)	0.96657 (19)	0.24156 (17)	0.0546 (8)	
C9	−0.1249 (6)	1.0343 (2)	0.2327 (2)	0.0692 (10)	
H9	−0.0909	1.0754	0.2012	0.083*	
C10	−0.2936 (6)	1.0428 (2)	0.2704 (2)	0.0818 (11)	
H10	−0.3687	1.0899	0.2644	0.098*	
C11	−0.3493 (5)	0.9826 (2)	0.3159 (2)	0.0739 (10)	
H11	−0.4636	0.9885	0.3397	0.089*	
C12	−0.3019 (5)	0.84972 (19)	0.37466 (16)	0.0521 (8)	
H12	−0.4259	0.8548	0.3919	0.062*	
C13	0.2168 (7)	1.0121 (3)	0.1557 (3)	0.1097 (16)	
H13A	0.1166	1.0151	0.1103	0.165*	
H13B	0.3447	0.9980	0.1411	0.165*	
H13C	0.2279	1.0631	0.1813	0.165*	
C14	0.3338 (5)	0.76176 (16)	0.61150 (16)	0.0486 (7)	
C15	0.3942 (4)	0.82084 (16)	0.56241 (16)	0.0449 (7)	
C16	0.5794 (4)	0.86010 (17)	0.58366 (16)	0.0473 (7)	
C17	0.7038 (5)	0.8378 (2)	0.65066 (18)	0.0590 (8)	
H17	0.8286	0.8626	0.6639	0.071*	
C18	0.6446 (5)	0.7784 (2)	0.69882 (19)	0.0663 (9)	
H18	0.7296	0.7642	0.7439	0.080*	
C19	0.4630 (6)	0.74141 (19)	0.68010 (19)	0.0642 (9)	
H19	0.4237	0.7024	0.7127	0.077*	
C20	0.1429 (5)	0.72198 (18)	0.59146 (19)	0.0562 (8)	
H20	0.0997	0.6862	0.6263	0.067*	
C21	0.8030 (5)	0.9613 (2)	0.5508 (2)	0.0764 (10)	
H21A	0.8104	0.9855	0.6010	0.115*	
H21B	0.8090	1.0022	0.5127	0.115*	
H21C	0.9142	0.9251	0.5508	0.115*	

### Atomic displacement parameters (Å^2^)

**Table:**

	*U*^11^	*U*^22^	*U*^33^	*U*^12^	*U*^13^	*U*^23^
O1	0.0634 (14)	0.0594 (14)	0.0767 (16)	0.0037 (11)	0.0238 (12)	0.0013 (11)
O2	0.0888 (18)	0.0737 (16)	0.0857 (17)	−0.0129 (13)	0.0431 (15)	0.0000 (13)
O3	0.0569 (14)	0.0611 (14)	0.0566 (13)	−0.0124 (10)	−0.0058 (11)	0.0136 (10)
O4	0.0603 (14)	0.0705 (14)	0.0561 (13)	−0.0203 (11)	0.0054 (10)	0.0077 (11)
N1	0.0526 (15)	0.0518 (15)	0.0452 (14)	−0.0090 (12)	0.0067 (12)	−0.0050 (12)
N2	0.0538 (16)	0.0436 (13)	0.0595 (17)	−0.0028 (11)	0.0103 (13)	0.0042 (12)
N3	0.077 (2)	0.075 (2)	0.079 (2)	−0.0199 (16)	0.0192 (17)	−0.0153 (16)
C1	0.064 (2)	0.059 (2)	0.065 (2)	−0.0197 (17)	0.0220 (18)	−0.0158 (17)
C2	0.065 (2)	0.0572 (19)	0.065 (2)	−0.0172 (17)	0.0225 (18)	−0.0119 (17)
C3	0.079 (2)	0.0536 (19)	0.078 (2)	−0.0128 (17)	0.0222 (19)	0.0057 (17)
C4	0.073 (2)	0.064 (2)	0.075 (3)	−0.0230 (19)	0.024 (2)	−0.0108 (19)
C5	0.071 (2)	0.066 (2)	0.073 (2)	−0.0255 (18)	0.021 (2)	−0.0150 (19)
C6	0.0500 (18)	0.0562 (18)	0.0426 (16)	0.0026 (14)	0.0048 (13)	−0.0032 (14)
C7	0.0541 (18)	0.0462 (17)	0.0439 (17)	−0.0047 (14)	0.0048 (14)	−0.0100 (14)
C8	0.0592 (19)	0.0589 (19)	0.0462 (18)	−0.0128 (16)	0.0097 (15)	−0.0099 (15)
C9	0.089 (3)	0.060 (2)	0.056 (2)	−0.0070 (19)	0.0038 (19)	0.0060 (16)
C10	0.086 (3)	0.070 (2)	0.090 (3)	0.019 (2)	0.016 (2)	0.015 (2)
C11	0.070 (2)	0.074 (2)	0.081 (3)	0.0126 (19)	0.0208 (19)	0.005 (2)
C12	0.0511 (18)	0.064 (2)	0.0417 (17)	−0.0053 (16)	0.0087 (14)	−0.0109 (15)
C13	0.138 (4)	0.103 (3)	0.104 (3)	−0.029 (3)	0.070 (3)	0.011 (3)
C14	0.0553 (19)	0.0439 (16)	0.0463 (18)	0.0002 (14)	0.0066 (14)	0.0010 (14)
C15	0.0475 (17)	0.0463 (16)	0.0398 (16)	0.0043 (13)	0.0035 (13)	0.0029 (13)
C16	0.0494 (18)	0.0504 (17)	0.0429 (17)	−0.0016 (14)	0.0092 (14)	−0.0024 (14)
C17	0.0512 (19)	0.070 (2)	0.054 (2)	0.0026 (16)	0.0015 (15)	−0.0093 (16)
C18	0.073 (2)	0.072 (2)	0.0492 (19)	0.0068 (19)	−0.0065 (17)	0.0083 (17)
C19	0.084 (3)	0.0557 (19)	0.051 (2)	0.0035 (18)	0.0050 (18)	0.0115 (16)
C20	0.071 (2)	0.0481 (17)	0.052 (2)	−0.0018 (15)	0.0177 (17)	0.0073 (15)
C21	0.061 (2)	0.080 (2)	0.087 (3)	−0.0251 (19)	0.0094 (19)	0.002 (2)

### Geometric parameters (Å, °)

**Table:**

O1—C7	1.340 (3)	C8—C9	1.366 (5)
O1—H1	0.8200	C9—C10	1.400 (5)
O2—C8	1.383 (4)	C9—H9	0.9300
O2—C13	1.428 (4)	C10—C11	1.369 (5)
O3—C15	1.350 (3)	C10—H10	0.9300
O3—H3	0.8200	C11—H11	0.9300
O4—C16	1.369 (3)	C12—H12	0.9300
O4—C21	1.421 (4)	C13—H13A	0.9600
N1—C12	1.267 (4)	C13—H13B	0.9600
N1—C1	1.423 (4)	C13—H13C	0.9600
N2—C20	1.288 (4)	C14—C15	1.407 (4)
N2—C2	1.410 (4)	C14—C19	1.410 (4)
N3—C5	1.397 (4)	C14—C20	1.436 (4)
N3—C1	1.400 (4)	C15—C16	1.402 (4)
C1—C2	1.408 (5)	C16—C17	1.382 (4)
C2—C3	1.401 (4)	C17—C18	1.397 (4)
C3—C4	1.377 (5)	C17—H17	0.9300
C3—H3A	0.9300	C18—C19	1.360 (5)
C4—C5	1.359 (5)	C18—H18	0.9300
C4—H4	0.9300	C19—H19	0.9300
C5—H5	0.9300	C20—H20	0.9300
C6—C11	1.389 (4)	C21—H21A	0.9600
C6—C7	1.416 (4)	C21—H21B	0.9600
C6—C12	1.459 (4)	C21—H21C	0.9600
C7—C8	1.406 (4)		
			
C7—O1—H1	109.5	C10—C11—H11	120.0
C8—O2—C13	118.3 (3)	C6—C11—H11	120.0
C15—O3—H3	109.5	N1—C12—C6	121.7 (3)
C16—O4—C21	117.5 (2)	N1—C12—H12	119.1
C12—N1—C1	118.0 (3)	C6—C12—H12	119.1
C20—N2—C2	123.2 (3)	O2—C13—H13A	109.5
C5—N3—C1	118.6 (3)	O2—C13—H13B	109.5
N3—C1—C2	120.3 (3)	H13A—C13—H13B	109.5
N3—C1—N1	120.9 (3)	O2—C13—H13C	109.5
C2—C1—N1	118.7 (3)	H13A—C13—H13C	109.5
C3—C2—C1	118.8 (3)	H13B—C13—H13C	109.5
C3—C2—N2	124.9 (3)	C15—C14—C19	119.5 (3)
C1—C2—N2	116.3 (3)	C15—C14—C20	120.3 (3)
C4—C3—C2	120.2 (4)	C19—C14—C20	120.3 (3)
C4—C3—H3A	119.9	O3—C15—C16	117.7 (2)
C2—C3—H3A	119.9	O3—C15—C14	122.9 (3)
C5—C4—C3	120.9 (3)	C16—C15—C14	119.4 (3)
C5—C4—H4	119.5	O4—C16—C17	126.2 (3)
C3—C4—H4	119.5	O4—C16—C15	114.2 (2)
C4—C5—N3	121.1 (3)	C17—C16—C15	119.6 (3)
C4—C5—H5	119.4	C16—C17—C18	120.8 (3)
N3—C5—H5	119.4	C16—C17—H17	119.6
C11—C6—C7	118.9 (3)	C18—C17—H17	119.6
C11—C6—C12	118.4 (3)	C19—C18—C17	120.3 (3)
C7—C6—C12	122.7 (3)	C19—C18—H18	119.9
O1—C7—C8	117.2 (3)	C17—C18—H18	119.9
O1—C7—C6	122.3 (3)	C18—C19—C14	120.4 (3)
C8—C7—C6	120.5 (3)	C18—C19—H19	119.8
C9—C8—O2	125.2 (3)	C14—C19—H19	119.8
C9—C8—C7	118.7 (3)	N2—C20—C14	121.1 (3)
O2—C8—C7	116.1 (3)	N2—C20—H20	119.5
C8—C9—C10	120.9 (3)	C14—C20—H20	119.5
C8—C9—H9	119.5	O4—C21—H21A	109.5
C10—C9—H9	119.5	O4—C21—H21B	109.5
C11—C10—C9	120.7 (3)	H21A—C21—H21B	109.5
C11—C10—H10	119.6	O4—C21—H21C	109.5
C9—C10—H10	119.6	H21A—C21—H21C	109.5
C10—C11—C6	120.1 (3)	H21B—C21—H21C	109.5

### Hydrogen-bond geometry (Å, °)

**Table:**

*D*—H···*A*	*D*—H	H···*A*	*D*···*A*	*D*—H···*A*
O1—H1···N1	0.82	1.96	2.683 (3)	146
O3—H3···N2	0.82	1.85	2.568 (3)	146
